# Trends in CD4+ Cell Counts, Viral Load, Treatment, Testing History, and Sociodemographic Characteristics of Newly Diagnosed HIV Patients in Osaka, Japan, From 2003 through 2017: A Descriptive Study

**DOI:** 10.2188/jea.JE20210150

**Published:** 2023-05-05

**Authors:** Fumiko Kagiura, Ryota Matsuyama, Dai Watanabe, Yuuki Tsuchihashi, Kazuhiko Kanou, Takuri Takahashi, Yusuke Matsui, Masayuki Kakehashi, Tomimasa Sunagawa, Takuma Shirasaka

**Affiliations:** 1 Graduate School of Biomedical and Health Sciences, Hiroshima University, Hiroshima, Japan; 2 Faculty of Nursing, Hiroshima International University, Hiroshima, Japan; 3 AIDS Medical Center, National Hospital Organization Osaka National Hospital, Osaka, Japan; 4 Infectious Disease Surveillance Center, National Institute of Infectious Diseases, Tokyo, Japan

**Keywords:** HIV, CD4, epidemiology, sociodemographic characteristics, descriptive study

## Abstract

**Background:**

The CD4 cell count of patients during diagnosis and distribution of CD4 cell counts in the patient population are important to understand infection-diagnosis interval and incidence rate of human immunodeficiency virus (HIV) infection, respectively. However, this information has not been published in Japan. This study aimed to describe the change in CD4 cell count trends and clarify the change in patients’ characteristics in association with the CD4 cell count information.

**Methods:**

A descriptive study was conducted to analyze the medical records of patients with HIV who visited one of the largest acquired immunodeficiency syndrome (AIDS) core hospitals in western Japan. The basic characteristics, CD4 cell counts, viral loads, and diagnosis-treatment intervals between the first (2003–2010) and second (2011–2017) halves of the study duration were compared.

**Results:**

The distribution of CD4 cell counts significantly changed between 2003–2010 and 2011–2017 (χ^2^ = 20.42, *P* < 0.001). The proportion of CD4 cell count <200 cells/mm^3^ increased (38.8% in 2003 to 45.9% in 2017), whereas CD4 cell count ≥500 cells/mm^3^ decreased (19.4% in 2003 to 12.2% in 2017). Moreover, the distributions of age groups, history of HIV screening test, patient outcomes, HIV viral load, and diagnosis-treatment interval also significantly changed (χ^2^ = 25.55, *P* < 0.001; χ^2^ = 8.37, *P* = 0.015; χ^2^ = 6.07, *P* = 0.014; χ^2^ = 13.36, *P* = 0.020; χ^2^ = 173.76, *P* < 0.001, respectively).

**Conclusion:**

This study demonstrated the fundamental trends of the HIV epidemic in Osaka, Japan between 2003–2010 and 2011–2017 and indicated that the incidence rate of HIV was decreasing in Japan.

## INTRODUCTION

The reported number of new cases of human immunodeficiency virus (HIV) infection in Japan reached its peak in 2013 and then has been decreasing gradually,^1^ suggesting a decrease in the incidence rate of recent HIV infection. However, the diagnosis of HIV infection gets delayed largely because of its clinical and epidemiological features, such as: (i) mild/nonspecific symptoms (eg, fever, rash, lymphadenopathy, etc) or even no symptom during the acute stage among patients,^2^ (ii) asymptomatic HIV infection until clear symptoms are developed at the acquired immunodeficiency syndrome (AIDS) stage,^3^ and (iii) less number of patients with HIV who get the serological tests done in its latent period.^4^ Therefore, this delay in diagnosis should be considered to accurately understand the epidemic trend of HIV infections.

It is important to assess the CD4+ T-cell count (CD4 cell count) of patients with HIV/AIDS because of their close association with disease progression.^5^ In the natural history of HIV infection, the HIV targets immune system cells, mainly CD4 cells, and induces their apoptosis.^2^ Gradually, it impairs the host immune system, causing immunodeficiency,^6^ which eventually leads to opportunistic diseases.^7^ When a patient with HIV infection develops one of the 23 specified opportunistic diseases, he/she is classified as a patient with AIDS in Japan.^8^ The average period between the infection and development of AIDS has been reported to be 8 years.^9^^,^^10^ Moreover, the mean time from symptom onset to death was found to be 2 years.^11^ Thus, the degree of decrease in CD4 cell count reflects the periods of occurrence of these events. Hence, in theory, knowledge on the rate of decrease in CD4 cells and its distribution would help in drawing inference about the time of infection onset.^12^

Nevertheless, the national HIV surveillance in Japan did not include individual CD4 cell counts until 2019,^13^ although those have been regularly tested in some hospitals.^14^ Therefore, the trend in HIV infection cannot be appropriately interpreted from Japan’s surveillance data due to the lack of comprehensive time-series information on the CD4 cell counts. However, such information is available at the hospital level. Hence, it is worth investigating the hospital-level CD4 cell count data, especially from hospitals that stock long-term series data covering a large group of patients with HIV/AIDS. In Japan, regional AIDS core hospitals are designated by the Ministry of Health, Labour and Welfare to provide effective HIV/AIDS care. The CD4 cell count data from those hospitals may shed light on the hidden trend of HIV infection when interpreted together with patients’ information.

The present study collected and summarized data from an AIDS core hospital in Japan, aiming to (i) describe the trend of CD4 cell count groups and (ii) clarify the epidemiological change in patients’ characteristics for the interpretation of the HIV infection trend in Japan in association with the CD4 information. There are few studies summarizing characteristics of CD4 cell count among newly diagnosed patients with HIV in Japan. This is the first large-scale population-based epidemiological study using CD4 cell count data and could aid in understanding the HIV/AIDS epidemic in Japan.

## METHODS

### Design and data source

This was a descriptive study of CD4 cell count data among patients with HIV at one of the largest AIDS core hospitals in western Japan. A part of the medical records data of patients who were diagnosed as HIV carriers during 2003–2017 was used. The data contained sociodemographic characteristics and blood test results at the first visit, as well as patient outcomes and day of antiretroviral therapy (ART) initiation. The patient outcomes and the day of ART initiation were assessed in 2018. The sociodemographic characteristics data contained the following information: the year of their first visit, age at that time, sex, nationality, risk groups of HIV infection (eg, men who have sex with men [MSM], heterosexuals, injecting drug users), clinical stage of HIV infection (asymptomatic carrier [AC], recent HIV infection, acute HIV infection, and AIDS, as described later), and history of HIV screening. The blood test results contained the CD4 cell count and HIV viral load. The patient outcomes were classified into two categories (ie, continuously taking treatment in the hospital or deceased). The day of initiation of ART was only available since 2006. Patients who had already undergone ART in any other medical service were excluded from the study.

The clinical stage of HIV infection was classified into four stages: acute HIV infection, recent HIV infection, AC stage, and AIDS. Acute HIV infection was defined as the state with symptoms, such as high fever, rash, and lymphadenopathy, with a negative or intermediate reaction in Western blotting but a positive result in polymerase chain reaction during the HIV-1 infection diagnosis. Recent HIV infection was defined as being infected with HIV in the past 6 months with a ≤6-month interval from the last negative HIV test until the first positive HIV test. The AC stage was defined as the state wherein no symptoms and had a >6-month interval from the last negative HIV test until the first positive HIV test. Lastly, AIDS was defined as an HIV infection with at least 1 of the 23 opportunistic diseases.^8^

### Statistical analysis

We summarized all data descriptively and calculated the proportions of the components in each category for each year. To clarify the change in trend of the yearly proportions, we divided the observed time periods into two parts: the first period, from 2003 through 2010, and the second period, from 2011 through 2017. This dichotomization was based on the change in the number of new HIV cases in the observed data (see Results section). The data of 2003 and 2004 for the variable “day of initiation of ART” were excluded from the analysis because the data were not available. To statistically determine whether a significant difference existed between the two periods, chi-square or Fisher’s exact test (when including a small sample category) was employed. A *P* value <0.05 was considered statistically significant. The statistical software R version 3.5.1 (R Foundation for Statistical Computing, Vienna, Austria)^15^ and its package stats and ggplot2^16^ were used for data analysis and graphical presentations.

### Ethical consideration

The opt-out method was employed to inform and receive consent from the participants, assuring that their data were anonymous and unidentified. The information on the participation for this research was presented on the websites of the National Institute of Infectious Diseases and the National Hospital Organization Osaka National Hospital. For patient anonymity, the data had no individual identifiable information (eg, personal name, medical ID) and no link to original medical records. The Ethical Committee for Epidemiology approved the study at all participating sites (National Institute of Infectious Diseases, No. 1160; Hiroshima University, No. E-2199; National Hospital Organization Osaka National Hospital, No 18099).

## RESULTS

### Sociodemographic characteristics of newly diagnosed HIV patients

**Figure 1.**
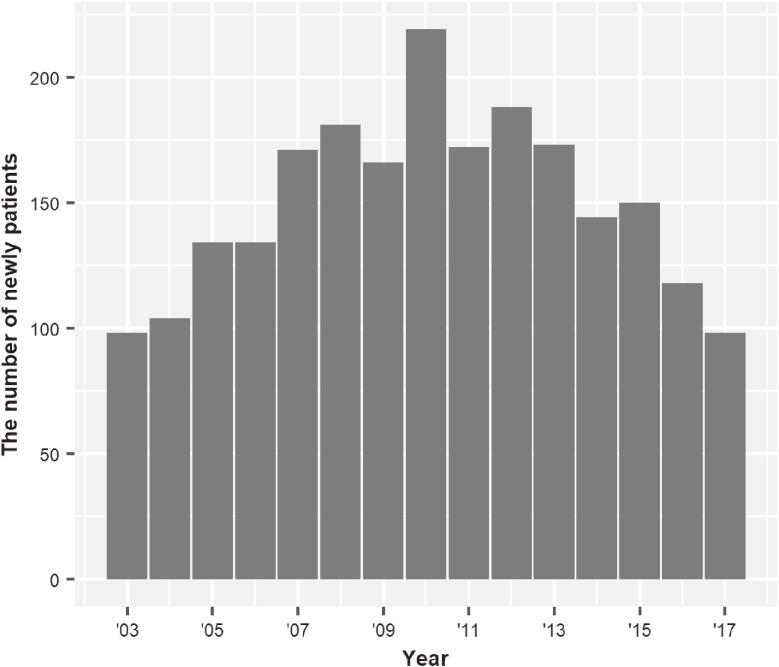
The number of new patients in 2003–2017

The data contained medical records of 2,250 patients from 2003 to 2017 (2,189 men and 61 women). Table 1 shows the characteristics of the patients. Figure 1 shows that the largest number of newly reported HIV infections was in 2010 (*N* = 211) and the second largest was in 2012 (*N* = 195). From 2003 through 2010, the number of cases increased, and after 2010, it decreased. Therefore, we dichotomized the data before and after 2010 to clarify the change of the trend.

**Table 1.** The characteristics of the patients

			*N*	%
Sex	Man	Japanese	2,111	93.8
		Non-Japanese	78	3.5
	Woman	Japanese	51	2.3
		Non-Japanese	10	0.4
Age at the first visit, years		15–19	15	0.7
		20–29	581	25.8
		30–39	819	36.4
		40–49	491	21.8
		50 and older	344	15.3
HIV risk groups		Men who have sex with men	1,845	82.0
		Heterosexuals	328	14.6
		Injecting drug users	5	0.2
		Other	72	3.2
Stage of HIV infection		Acute HIV infection	185	8.2
		Recent HIV infection	188	8.4
		AC stage	1,342	59.6
		AIDS	533	23.7
		Missing value	2	0.1

AC, asymptomatic carrier; AIDS, acquired immunodeficiency syndrome; HIV, human immunodeficiency virus.

The result of the chi-square test showed that the distribution of the age was significantly different between the first and second periods (χ^2^ = 25.55, *P* < 0.001). The proportion of cases in the 30–39-year age group reached its peak in 2006 (47.0%) and then gradually decreased (33.7% in 2017, Figure 2A). In contrast, the proportion of cases in the 40–49-year age group increased from 17.3% in 2003 to 26.5% in 2017. Detailed data in this regard are provided in eTable 1.

**Figure 2.**
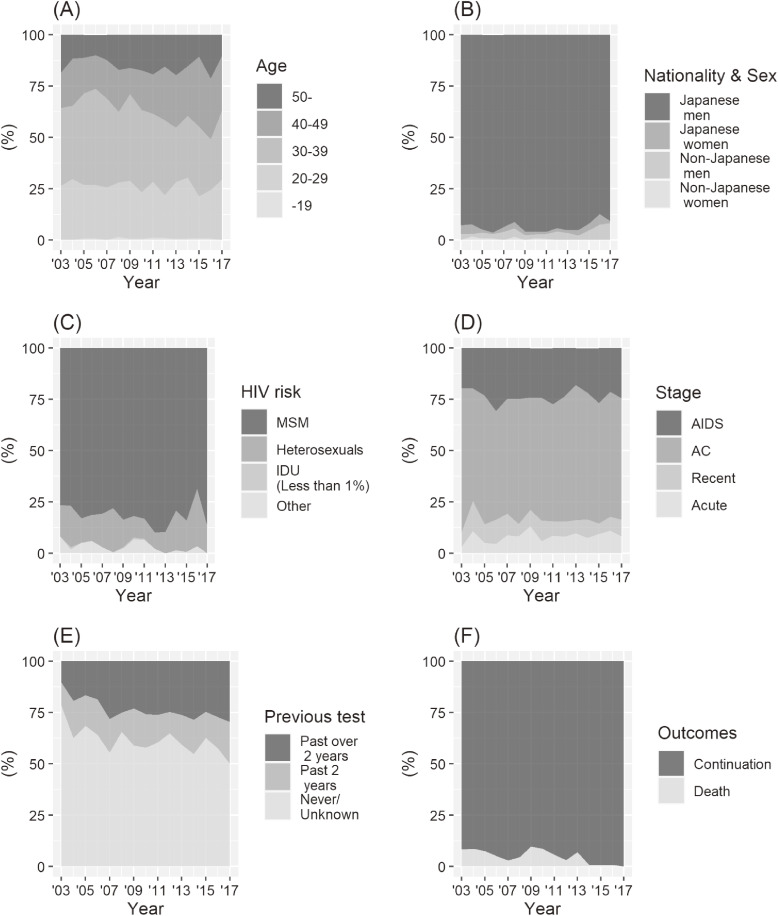
Temporal change in the demographics of new patients in 2003–2017: (A) Age, (B) nationality and sex, (C) HIV risk, (D) HIV stage, (E) previous HIV screening test, and (F) patient outcomes (continuation: continuously taking treatment in the hospital, death: dead)

Figure 2B shows temporal change of the nationality and sex among the new patients. The distributions of nationality and sex did not significantly change between the first and second periods (Fisher’s exact test; *P* = 0.201). Most of the new patients were Japanese men (87.3–95.9%) throughout the study period.

The distribution of the HIV risk groups did not change between the first and second terms (Fisher’s exact test; *P* = 0.281). The largest group in each year was consistently MSM (68.6–89.9%, Figure 2C). The proportion of injection drug users was consistently small (range, 0.0–1.0%).

The proportion of the HIV stage did not change significantly in the first and second periods (χ^2^ = 2.61, *P* = 0.456). The AC stage, which ranged from 54.8% in 2009 to 70.4% of cases in 2003 (Figure 2D), was consistently the most common. Acute HIV infection and recent HIV infection ranged from 3.1% in 2003 to 13.3% in 2009 and 5.3% in 2015 to 14.7% in 2004.

Figure 2E shows the proportion of patients who had a history of HIV testing, which significantly changed between the first and second periods (χ^2^ = 8.37, *P* = 0.015). The proportion of patients who never had a HIV screening test previously but was detected as HIV positive in subsequent visits decreased from 78.6% in 2003 to 50.0% in 2017 (Figure 2E).

Lastly, Figure 2F shows the patient outcomes of newly diagnosed HIV patients who visited the hospital, the proportions of which was significantly different between the first and second periods (χ^2^ = 6.07, *P* = 0.014). The proportion of cases resulting in death decreased from 8.2% in 2003 to 0.0% in 2017.

### Trends of CD4 cell count and viral load in newly diagnosed HIV patients

**Figure 3.**
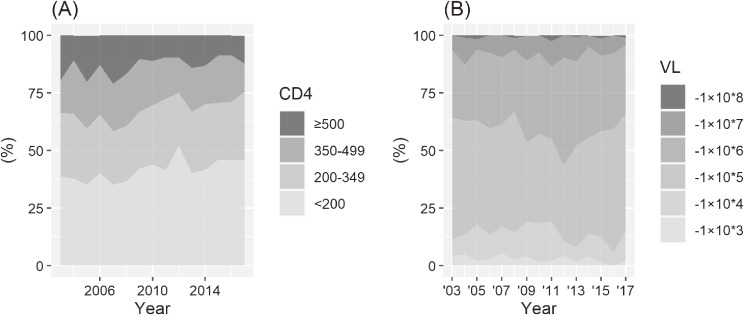
Temporal change in (A) distribution of CD4 cell count at the first visit and (B) distribution of HIV viral load: In (A), the proportion of the ≥500 CD4 cell count decreased.

The CD4 cell count was classified into four categories in accordance with the treatment guidelines for HIV infections,^17^ as this style of classification is accepted globally.^18^ The grouping was performed using the number of CD4+ T-cells in 1 mm^3^ of patient’s blood, and the four categories were as follows: <200, 200–349, 350–499, and ≥500 cells/mm^3^. The proportion of the groups had significantly changed between the first and second periods (χ^2^ = 20.42, *P* < 0.001). The proportion of CD4 cell count ≥500 cells/mm^3^ group, which is the group with the best immunological condition, had decreased from 19.4% in 2003 to 12.2% in 2017 (Figure 3). The proportion of CD4 cell count 350–499 cells/mm^3^, the second-best immunological condition group, had also decreased from the first to the second period. The proportion of the CD4 cell count <200 cells/mm^3^ group had increased from 38.8% in 2003 to 45.9% in 2017 and was the most frequently encountered group.

The amount of HIV-RNA (viral load) in newly diagnosed HIV patients changed between the first and second periods (χ^2^ = 13.36, *P* = 0.020). The proportion of three low viral load groups (ie, <1 × 10^5^ copies/mL) slightly decreased, whereas the proportion of the viral load group between 1 × 10^6^ and 1 × 10^7^ copies/mL had an increasing trend, ranging from 24.3% in 2004 to 46.8% in 2012 (Figure 3). The most common group regarding the viral load was consistently between 1 × 10^5^ and 1 × 10^6^ copies/mL, except in 2009 and 2012.

### Days between the first visit and starting ART of newly diagnosed HIV patients

**Figure 4.**
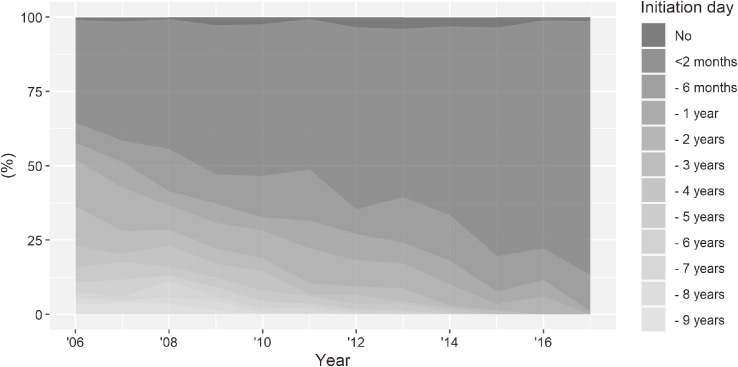
Temporal change (2006–2017) of the interval distribution from diagnosis to the start of ART

The proportion of patients who started ART within 2 months after the first visit had increased from 34.7% in 2006 to 85.6% in 2017 (Figure 4) and was significantly different between the first and second periods (χ^2^ = 173.76, *P* < 0.001).

## DISCUSSION

This study described the trend of HIV infection in Japan using the medical records data from one of the largest AIDS core hospitals in western Japan. The proportions of basic characteristics in the present data and the national surveillance data were similar. Therefore, it was considered that the data used in this study reflected the trend of HIV infections in Japan. The analysis clarified that, during the second period, the proportion of CD4 cell count ≥500 cells/mm^3^ category decreased and that of the CD4 cell count <200 cells/mm^3^ category increased when compared to that in the first period. Moreover, there were significant differences in age, history of HIV screening test, patient outcomes, HIV viral load, and the initiation of ART between the two periods. These results indicate that the incidence rate of HIV is decreasing in Japan.

Although the data were collected only from one AIDS core hospital, its share in the reported number of patients in the national HIV surveillance^1^ during 2003–2017 was large (10.5% on average). In addition, the distribution of age and its trend in time-dependent change were also very similar to those observed in the national HIV surveillance data.^1^ Moreover, the distributions of nationality and risk groups were similar to those in the national data.^1^ While there was a comparatively higher proportion of Japanese males (93.6% on average) in the nationality data of the present study than that in national HIV surveillance (85.4% on average) was observed, the domination of Japanese males and the subsequent order of other categories showed a large similarity. Even though the proportions of MSM in the risk group has been reported to be about 70% in Japan’s national surveillance data,^1^ it has been pointed out that those were underestimated values due to the hesitancy in the response of MSM.^19^ Hence, we believe the results of the present study largely reflect the nationwide trend of HIV infection in Japan.

The results suggest that the significant decrease in the proportion of patients with the CD4 cell count ≥500 cells/mm^3^ and simultaneous increase in the proportion of patients with CD4 cell count <200 cells/mm^3^ were more likely caused by the decrease in HIV incidence rate than by the delay in diagnosis of HIV infection. It is supported by several pieces of evidence. First, decreasing incidence may be due to the shortening of time between the diagnosis and the start of ART (Figure 4). ART is known not only to stop the progression of the disease^20^ but also cut the risk of transmission.^21^ Second, the decline in the proportion of patients with CD4 cell count ≥500 cells/mm^3^, and a gradational decline of the proportions of patients in the middle stages (CD4 cell count 350–499 and 200–349 cells/mm^3^) suggest that the stage of the entire HIV carrier population to some extent shifted to a more progressed stage. The proportion of CD4 count <200 cells/mm^3^ was consequently increased. Third, the proportion of those who had more than one HIV screening test increased among newly diagnosed HIV patients (Figure 2E). Similarly, a significant increase in the rate of voluntary HIV testing in Osaka Prefecture^1^^,^^22^ was observed between 2003 and 2017 (Jonckheere-Terpstra test, *P* = 0.040). Frequent conduct of HIV screening tests and increased testing of newly infected patients have been reported to impact the decrease in the incidence rate of HIV infection.^1^^,^^23^ Fourth, the rate of acute and recent HIV infections among those who voluntarily got tested using blood samples have been reported to have decreased in Osaka,^24^ indicating a decrease in the incidence rate as well. The above evidence strengthens the possibility that the incidence rate of HIV infection has been decreasing in Japan.

The significant differences in age distribution and in HIV viral load distribution between the two periods are probably associated with the observed differences in the CD4 cell count distribution. Low CD4 cell count has been reported to be associated with older age.^25^^,^^26^ In addition, CD4 cell count has been known to be inversely correlated to HIV viral load.^27^ Therefore, the increase in age and in HIV viral load of the present study may reflect the increased trend of the proportion of patients with <200 cells/mm^3^ CD4 cell counts.

This study has a few limitations. First, all the records created were based solely on the answers of the patients. Thus, it might not reflect true information. Some of the information (eg, the testing experience) could contain recall bias. Second, the data were collected from only one regional AIDS core hospital. Therefore, the results might not be accurately generalizable to the whole Japanese population. Third, the change of proportions in patient outcomes was affected not only by the reduction in mortality due to the spread of ART,^28^^,^^29^ but also probably by the censoring.

## SUPPLEMENTARY MATERIAL

eTable 1. Supplemental data table
