# Supplementary material for: Trends in CD4+ Cell Counts, Viral Load, Treatment, Testing History, and Sociodemographic Characteristics of Newly Diagnosed HIV Patients in Osaka, Japan, From 2003 through 2017: A Descriptive Study
Source: J Epidemiol. 2023 May 5;33(5):256–61. doi: 10.2188/jea.JE20210150 (PMC10043158; doi:10.2188/jea.JE20210150)
Supplement: Supplementary file 1 [file je-33-256-s001.pdf]

eTable 1. Supplemental data table

	Percentages in each year															Numbers		
	'03	'04	'05	'06	'07	'08	'09	'10	'11	'12	'13	'14	'15	'16	'17	'03–'10	'11–'17	p-value
**The number of new patients in 2003–2017 (used in Figure 1)**																		
	98	104	134	134	171	181	166	219	172	188	173	144	150	118	98	–	–	–
**Temporal change in the demographics of new patients in 2003–2017 (Figure 2)**																		
**Age, years (A)**																		
≤19	0.0	0.0	0.7	0.7	0.0	1.7	0.6	0.5	1.2	1.1	0.6	0.7	0.7	0.8	0.0	6	9	< 0.001
20–29	26.5	29.8	26.1	26.1	25.7	26.5	28.3	22.8	27.3	20.7	27.7	29.9	20.7	23.7	29.6	266	315	(X^2 =
30–39	37.8	35.6	44.8	47.0	43.3	34.3	42.2	40.2	33.1	36.7	26.6	29.9	34.0	24.6	33.7	403	416	25.55)
40–49	17.3	23.1	17.2	16.4	18.7	20.4	12.7	19.2	19.2	26.1	25.4	24.3	34.0	29.7	26.5	176	315	
≥50	18.4	11.5	11.2	9.7	12.3	17.1	16.3	17.4	19.2	15.4	19.7	15.3	10.7	21.2	10.2	137	207	
**Nationality and sex (B)**																		
Japanese men	92.9	92.3	94.8	96.3	93.6	91.2	95.8	95.9	95.9	94.1	95.4	95.1	92.0	87.3	90.8	927	1184	0.201
Japanese women	4.1	4.8	1.5	0.7	2.3	3.3	1.8	1.4	1.2	1.6	1.2	2.8	3.3	5.1	1.0	25	26	(Fisher's
Non-Japanese men	3.1	1.0	3.0	2.2	4.1	3.9	2.4	1.8	2.3	4.3	3.5	2.1	4.7	7.6	8.2	29	49	exact
Non-Japanese women	0.0	1.9	0.7	0.7	0.0	1.7	0.0	0.9	0.6	0.0	0.0	0.0	0.0	0.0	0.0	7	3	test)
**HIV risk (C)**																		
MSM	76.5	76.9	82.8	81.3	80.7	77.9	83.7	81.7	83.1	89.9	89.6	79.2	84.0	68.6	86.7	793	1052	0.281
Heterosexuals	15.3	20.2	11.9	12.7	16.4	21.5	13.3	10.5	9.9	8.0	10.4	19.4	15.3	28.0	13.3	158	170	(Fisher's
IDU	0.0	1.0	0.0	0.0	0.0	0.0	0.6	0.9	0.6	0.0	0.0	0.0	0.0	0.0	0.0	2	3	exact
Other	8.2	1.9	5.2	6.0	2.9	0.6	2.4	6.8	6.4	2.1	0.0	1.4	0.7	3.4	0.0	35	37	test)
**HIV Stage (D)**																		
AIDS	19.4	19.6	23.1	30.6	24.6	24.9	24.1	24.2	27.3	23.4	17.9	21.5	26.7	21.2	24.5	238	295	0.456
AC	70.4	54.9	62.7	53.0	56.1	60.8	54.8	59.8	57.0	61.2	65.9	61.8	58.7	61.0	59.2	577	765	(X^2 =
Recent	7.1	14.7	9.0	11.9	10.5	6.1	7.8	10.0	7.0	7.4	6.4	9.0	5.3	6.8	8.2	92	96	2.61)
Acute	3.1	10.8	5.2	4.5	8.8	8.3	13.3	5.9	8.7	8.0	9.8	7.6	9.3	11.0	8.2	79	106	
**Previous HIV screening test (E)**																		
>2 years ago	10.2	19.2	16.4	18.7	28.1	24.9	22.9	25.6	26.2	24.5	26.0	28.5	24.7	27.1	29.6	208	331	0.015
Past 2 years	11.2	18.3	14.9	17.2	16.4	9.4	18.1	16.4	13.4	10.6	14.5	16.7	12.7	15.3	20.4	148	185	(X^2 =
Never/Unknown	78.6	62.5	68.7	64.2	55.6	65.7	59.0	58.0	60.5	64.9	59.5	54.9	62.7	57.6	50.0	632	746	8.37)

	Percentages in each year															Numbers		
	'03	'04	'05	'06	'07	'08	'09	'10	'11	'12	'13	'14	'15	'16	'17	'03–'10	'11–'17	p-value
**Patient outcomes (F)**																		
Continuation	91.8	91.4	92.4	94.8	97.0	95.4	90.2	91.3	94.3	96.9	93.1	99.2	99.2	99.1	100.0	727	1012	0.014 (X^2 = 6.07)
Death	8.2	8.6	7.6	5.2	3.0	4.6	9.8	8.7	5.7	3.1	6.9	0.8	0.8	0.9	0.0	50	40	
**Temporal change in the distribution of the CD4 cell count at the first visit and of the HIV viral load (Figure 3)**																		
**CD4 cell count at the first visit**																		
≥500	19.4	10.7	20.1	12.7	21.1	16.7	10.2	11.0	9.4	9.6	14.0	13.2	8.7	8.5	12.2	157	136	< 0.001 (X^2 = 20.42)
350–499	14.3	23.3	20.1	21.6	20.5	22.8	22.9	19.3	18.1	15.4	19.2	16.7	20.7	20.3	12.2	208	226	
200–349	27.6	28.2	24.6	25.4	23.4	23.9	24.7	25.7	31.0	22.9	26.7	28.5	24.7	25.4	29.6	247	335	
<200	38.8	37.9	35.1	40.3	35.1	36.7	42.2	44.0	41.5	52.1	40.1	41.7	46.0	45.8	45.9	374	562	
**HIV viral load**																		
-1×10*8	0.0	1.0	1.5	0.0	0.0	1.1	0.0	0.0	2.3	0.0	0.6	0.0	1.3	0.0	1.0	5	8	0.020 (X^2 = 13.36)
-1×10*7	6.1	11.7	4.5	7.5	9.4	5.1	10.8	7.3	11.1	9.6	10.5	4.9	7.3	7.6	3.1	77	101	
-1×10*6	29.6	24.3	30.6	32.8	29.2	27.0	35.5	35.3	31.6	46.8	36.6	39.6	32.7	33.1	29.6	296	456	
-1×10*5	53.1	49.5	45.5	46.3	44.4	52.2	34.3	39.0	35.7	33.0	44.2	41.7	46.0	53.4	51.0	452	526	
-1×10*4	7.1	8.7	15.7	10.4	11.7	11.8	15.1	17.0	17.0	6.4	6.4	9.7	11.3	5.9	13.3	117	140	
-1×10*3	4.1	4.9	2.2	3.0	5.3	2.8	4.2	1.4	2.3	4.3	1.7	4.2	1.3	0.0	2.0	37	28	
**Temporal change of the interval distribution from the diagnosis to the start of ART (Figure 4)**																		
No	–	–	–	0.8	1.3	0.6	2.6	2.5	0.6	3.3	3.8	2.9	3.5	0.9	1.1	8	29	< 0.001 (X^2 = 173.76)
–2 months	–	–	–	34.7	40.1	43.8	50.3	51.0	50.6	61.3	56.7	63.5	76.8	76.8	85.6	254	744	
–6 months	–	–	–	6.6	7.2	14.2	9.8	13.9	17.3	8.3	15.3	15.3	12.0	10.7	12.2	58	156	
–1 year	–	–	–	5.8	8.6	4.7	6.5	4.5	9.3	8.8	7.0	8.0	4.2	5.4	1.1	38	75	
–2 years	–	–	–	15.7	14.5	8.3	8.5	9.4	11.7	8.8	8.3	7.3	2.1	6.2	0.0	68	87	
–3 years	–	–	–	13.2	7.9	5.3	5.2	4.5	3.7	2.8	4.5	0.7	1.4	0.0	0.0	45	30	
–4 years	–	–	–	7.4	2.6	7.1	4.6	6.4	0.6	3.3	0.6	1.5	0.0	0.0	0.0	32	23	
–5 years	–	–	–	5.0	5.9	3.0	3.3	3.5	2.5	1.7	2.5	0.7	0.0	0.0	0.0	25	19	
–6 years	–	–	–	3.3	5.9	1.8	3.3	2.5	1.2	1.1	1.3	0.0	0.0	0.0	0.0	21	11	
–7 years	–	–	–	1.7	1.3	5.3	1.3	1.5	1.9	0.6	0.0	0.0	0.0	0.0	0.0	15	7	
–8 years	–	–	–	2.5	0.7	2.4	3.3	0.5	0.6	0.0	0.0	0.0	0.0	0.0	0.0	13	2	
–9 years	–	–	–	3.3	3.9	3.6	1.3	0.0	0.0	0.0	0.0	0.0	0.0	0.0	0.0	18	0	

AC, asymptomatic carrier; ART, antiretroviral therapy; AIDS, acquired immunodeficiency syndrome; HIV, human immunodeficiency virus; IDU, injecting drug users; MSM, men who have sex with men.
